# Influence of Tension and Tension Fluctuation on the Structure and Mechanical Properties of Polyester Fibers During the Spinning Process Based on Non-Contact Tension Detection

**DOI:** 10.3390/ma18173972

**Published:** 2025-08-25

**Authors:** Wanhe Du, Dongjian Zhang, Wei Fan, Shuzhen Yang, Xuehui Gan

**Affiliations:** 1 School of Intelligent Manufacturing and Control Engineering, Shanghai Polytechnic University, Shanghai 201209, China; whdu@sspu.edu.cn (W.D.); wfan@sandau.edu.cn (W.F.); szyang@sspu.edu.cn (S.Y.); 2 Tanac Automation Co., Ltd., Jiashan, Zhengjiang 314100, China; 3 School of Mechanical Engineering, Southeast University, Nanning 211189, China; 4 School of Engineering, Sanda University, Shanghai 201209, China; 5 College of Mechanical Engineering, Donghua University, Shanghai 201620, China

**Keywords:** fiber tension, tension fluctuation coefficient, fiber structural properties, fiber mechanical properties

## Abstract

The precise measuring and control of fiber tension are critically important for enhancing structural and mechanical properties in spinning processes, as tension directly influences orientation, crystallinity, and mechanical properties. However, current tension measurement methods primarily operate offline and lack real-time measuring capabilities. A non-contact fiber tension detection system is introduced to investigate the effects of draw tension and its uniformity on the structure and mechanical properties of polyester fibers. During experiments conducted at a spinning speed of 1200 m/min across different draw ratios, the non-contact system demonstrated strong agreement with the contact tension detector. The results showed that increasing the tension from 34 cN to 164 cN reduced the monofilament diameter from 39.61 µm to 20.35 µm. Simultaneously, the orientation factor nearly tripled, while crystallinity increased from 55.72% to 77.39%. Mechanical testing revealed a 50.96% improvement in breaking strength, rising from 1.57 to 2.37 cN/dtex, accompanied by a significant decrease in elongation at break from 275.55% to 34.95%. However, tension fluctuations, characterized by an average fluctuation coefficient increase from 4.51% to 18.18%, caused diameter inconsistency. These fluctuations also reduced the orientation factor by 10.78%, lowered crystallinity, and substantially deteriorated mechanical properties. These findings underscore the critical importance of real-time, online tension monitoring for ensuring polyester fiber quality and performance during production.

## 1. Introduction

The precise control of spinning tension is a critical determinant of product quality in the industrial manufacture of polyester fibers [1,2]. This parameter governs molecular orientation, crystallization kinetics, and defect formation during fiber formation, thereby directly dictating the ultimate mechanical properties of the fiber. The extent of applied tension significantly influences fiber diameter, crystallinity, and orientation, all fundamental factors underpinning key mechanical characteristics such as breaking strength and elongation at break [3,4,5]. By subjecting the polymer melt to tensile stress, the spinning process facilitates chain elongation and alignment, promoting molecular orientation and subsequent crystallization. Consequently, the final fiber performance, including breaking strength and elongation at break, is intrinsically linked to both the magnitude and fluctuation of tension exerted throughout spinning. Furthermore, effective tension control is paramount for minimizing structural defects, such as diameter variations and irregularities, thereby enhancing fiber homogeneity and overall quality [6,7]. The resultant fiber structure, encompassing crystalline and amorphous domains, is also profoundly shaped by the applied tension, with optimal levels fostering desirable crystallinity that enhances thermal stability, strength, and resistance to deformation [8].

While maintaining constant tension promotes fiber uniformity, tension fluctuations can precipitate significant quality issues. Such instabilities often arise from variations in polymer melt feed rates, fluctuations in spinning speed, or environmental disturbances like temperature and humidity changes [9,10,11]. These fluctuations manifest as inconsistent fiber diameters, leading to inhomogeneous mechanical properties along the fiber length. More severe variations can induce structural defects, such as weak points or excessive elongation, substantially compromising mechanical integrity and stability [12,13,14]. Critically, tension fluctuations detrimentally impact the crystallization behavior of polyester fibers. The mechanical stress applied during spinning governs chain alignment and orientation; irregular stress patterns due to tension instability disrupt this process, resulting in reduced crystallinity and weakened intermolecular interactions. Ultimately, this degradation diminishes fiber strength and thermal performance. Therefore, stringent control of tension fluctuations is essential to achieve consistent fiber quality and reliable mechanical properties [15,16].

The accurate, real-time measuring of spinning tension is indispensable for ensuring consistent fiber quality. Traditional measurement techniques predominantly rely on contact-based methods, such as load cells or roller-to-roller sensors. However, these approaches introduce inherent limitations: measurement inertia, potential damage to the fiber surface, and the requirement for frequent calibration and maintenance. Crucially, their suitability diminishes significantly in high-speed production lines exceeding 1000 m/min, where real-time responsiveness is paramount. This technological gap hinders the attainment of superior fiber structural homogeneity and performance consistency [17,18,19,20]. Recent advancements in non-contact measurement methodologies, particularly laser Doppler vibrometry and natural frequency extraction, offer promising alternatives [21,22]. Studies have demonstrated a strong correlation between the transverse vibration frequency of the fiber during spinning and the applied tension, enabling tension detection via real-time frequency monitoring [23,24]. Despite the emergence of these non-contact techniques, a systematic understanding of how both steady-state tension and dynamic tension fluctuations influence the structural evolution (diameter, orientation, and crystallinity) and mechanical properties of polyester fibers remains notably absent in the literature.

The structure evolution of polyester fibers is a crucial factor governing their mechanical properties. The extent of crystallization is determined by the rate of polymer chain alignment during spinning, which is itself modulated by process conditions, including tension, spinning speed, and cooling rate. Generally, higher tension promotes greater molecular alignment and crystallization, leading to stronger fibers with improved thermal resistance and dimensional stability [25,26,27,28]. However, excessive tension can induce over-orientation, potentially compromising fiber flexibility and inducing brittleness. Similarly, fiber morphology (encompassing cross-sectional shape and surface texture) is profoundly influenced by spinning parameters like tension and speed. Variations in these parameters can alter fiber cross-section, surface roughness, and overall appearance, features that critically influence functional properties such as moisture absorption, dye uptake, and abrasion resistance [29]. Consequently, elucidating the interplay between tension, tension fluctuation, and crystallization kinetics is vital for tailoring the microstructure of polyester fibers to specific application requirements.

The objectives of this study are to investigate the structural evolution of polyester fibers under various tension and tension fluctuation conditions and to explore the effects of these conditions on their mechanical properties. The changes in the structure and properties of polyester fibers at different tensions are analyzed via non-contact fiber tension measurement systems, X-ray diffraction, and mechanical testing. By gaining a comprehensive understanding of the structural evolution of polyester fibers under tension and tension fluctuations, we aim to reveal the mechanisms underlying the changes in their mechanical properties, which will facilitate the optimization of the spinning process and the production of high-quality polyester fibers. The results of this study are expected to promote the development of polyester fibers with excellent mechanical properties and uniform quality, broadening their application in various fields such as textiles and composites.

## 2. Experimental

### 2.1. Materials

This study employed polyethylene terephthalate (PET) chips manufactured by Sinopec Yizheng Chemical Fibre Co., Ltd. (Yizheng, China). The key properties of the PET chips were as follows: intrinsic viscosity is 0.675 dL/g, diethylene glycol (DEG) content is 1%, and carboxyl end group content is 21 mol/t.

### 2.2. Sample Preparation

**Figure 1:**
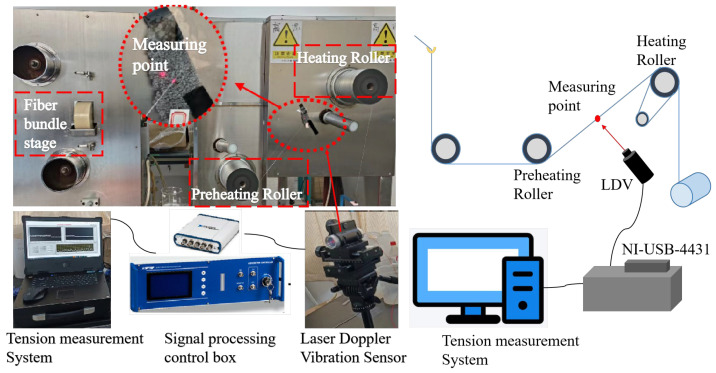
Schematic diagram of tension measurement in fiber preparation process.

Figure 1 illustrates the polyester fiber spinning process: Dry PET chips are fed into a screw extruder. The resulting melt is extruded through a spinneret to form nascent filaments. These filaments are then cooled. Subsequently, the filaments are bundled and oiled (finish applied) to form a cohesive strand. A godet roller, positioned directly beneath the oiling wheel, imparts tension to the fiber bundle during oiling, establishing the initial mechanical properties of the multifilament yarn. A preheating godet then provides initial thermal treatment to the fibers. The primary heating stage occurs on a heated godet, where the fiber bundle undergoes multiple wraps between this godet and a draw roll; this sustained heating modifies the internal fiber structure. The tension measurement point is located between the preheating godet and the heated godet. Finally, the fiber bundle is wound onto a package by a winding device. Differential samples were prepared by presetting specific tension parameters and fluctuation modes on the winder. The spinning speed was set at 1200 m/min.

### 2.3. Measurement of Fiber Tension

The experiment utilized a non-contact fiber tension measurement system to detect fiber tension in real-time under varying draw ratios and compared the results with those obtained from a contact tension measuring device (SZSH-200, Dongguan Sanliang Precision Measuring Instrument Co., Ltd., Dongguan, China). The specific operational procedure is as follows:

Step 1: The laser Doppler vibrometer system was employed to precisely focus on the fiber detection point (Figure 1). The system parameters were calibrated while the fiber was in a stationary state, ensuring that the signal-to-noise ratio of the vibration signal exceeded 15 dB before initiating dynamic measurements of the fiber vibration signal.

Step 2: A contact tension measuring device was used to perform ten repeated measurements under identical operating conditions, with the average value taken as the reference baseline. The measurement results from both methods were compared to validate the linear correlation coefficient of the non-contact system (R^2^ > 0.98).

Step 3: Gradient experimental points were established within the draw ratio range of λ ∈ [1.5, 3.0] (Δλ = 0.5). Data collection commenced after maintaining a stable production state for 300 s at each set of process parameters.

**Figure 2:**
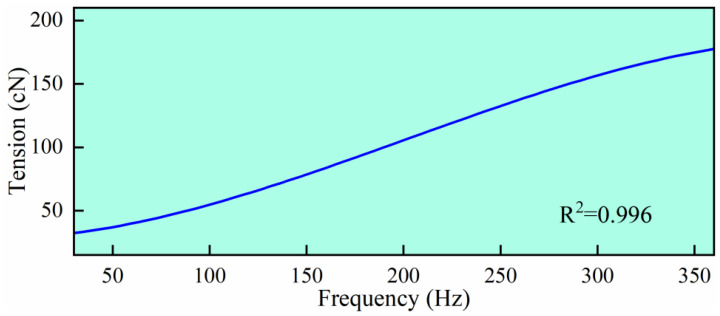
The relationship between fiber vibration frequency and fiber tension.

Step 4: Based on the author’s previous research results [30,31,32], the theoretical relationship between frequency and fiber tension is illustrated in Figure 2. Therefore, once the fiber’s natural frequency is detected during the spinning process, the fiber tension can be accurately measured.

**Figure 3:**
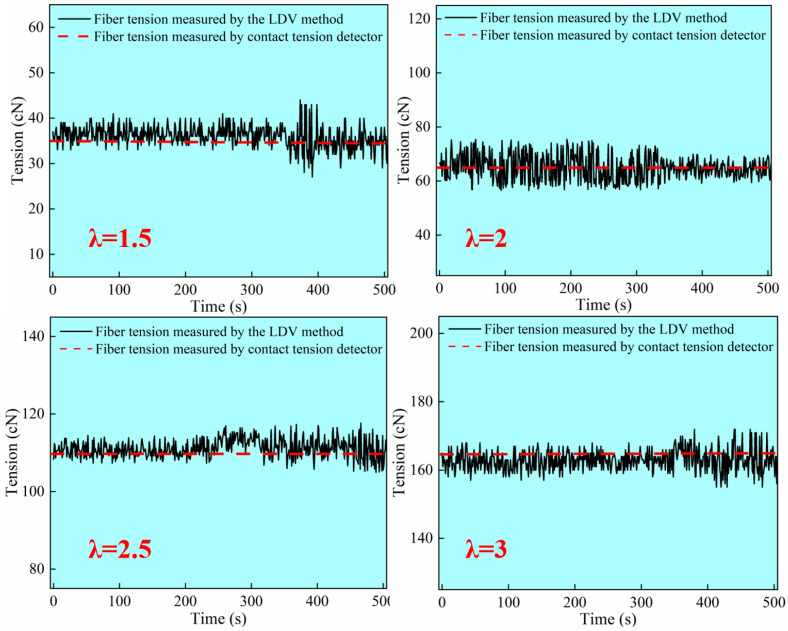
Comparison of fiber tension measurement results under different draft ratios.

The experimental process involved drawing ratios of 1.5, 2.0, 2.5, and 3.0, with the corresponding results from the contact tension measuring device being 34 cN, 65 cN, 111 cN, and 164 cN, respectively. A comparison of the results obtained from the non-contact fiber tension detection system with those from the contact tension measuring device revealed a strong agreement between the two methods. Notably, at a draw ratio of 3.0, the overall error compared to the contact method was maintained within 8%, as illustrated in Figure 3.

### 2.4. Testing and Characterization

The SCY-Ⅲ type sonic fiber orientation measuring instrument was employed to measure the sonic velocity of polyester fibers using the pulse method, resulting in a data table of fiber length and time [33]. By applying linear regression to the sonic velocity data, a linear relationship between fiber length and time was established, with the slope representing the sonic velocity of the fiber. The orientation factor was then calculated using Equation (1):

(1) $$f=1-C_{u}^{2}/C_{x}^{2},$$

Crystallinity characterization was performed using a Bruker D2PHASER one-dimensional X-ray diffractometer (Bruker, Billerica, MA, USA) [34]. The experimental conditions included CuKα radiation with a wavelength of 0.154 nm, a scanning angle of 5–70°, and a step width of 0.05°. Data processing was conducted using Peakfit software (V4.12), and the crystallinity was calculated according to Equation (2):

(2) $$C_{r}=\frac{S_{e}}{S_{e}+S_{n}}\times 100\%,$$

where $C_{r}$ represents the crystallinity of the fiber, $S_{e}$ denotes the area of the crystalline peaks, and $S_{n}$ indicates the area of the amorphous peaks.

The mechanical properties of the fibers, including the breaking strength and elongation at break, were evaluated using the YG020B (Xinfang Testing Instrument Equipment Co., Ltd., Changzhou, China) electronic single yarn strength and elongation testing system under varying tension and tension fluctuations [35].

### 2.5. Establishment of Tension Fluctuation Coefficient

**Figure 4:**
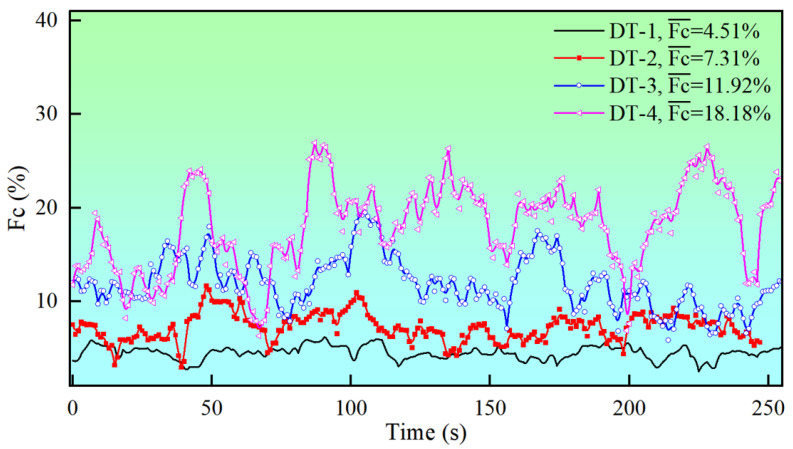
Tension fluctuation coefficient at a draft ratio of 1.5.

To investigate the effect of tension fluctuations on fiber properties, a tension fluctuation coefficient during the spinning process was established. Starting from a draw ratio of 1.5, the tension fluctuation conditions were altered by increasing the roller eccentricity and adjusting the frequency of the winder’s transverse shuttle. Four distinct operating conditions were defined as DT-1, DT-2, DT-3, and DT-4. The average tension fluctuation coefficients for these conditions over the respective time intervals were calculated as 4.51%, 7.31%, 11.92%, and 18.18%, as shown in Figure 4. The tension fluctuation coefficient was defined as

(3) Fc=∑n=in=i+9(xn−∑n=in=i+9xn/10)210∑n=in=i+9xn/10×100%,

where *x* represents the tension value at any given moment in time and $\overset{—}{F_{c}}$ denotes the average tension fluctuation coefficients.

## 3. Results and Discussion

### 3.1. Fiber Monofilament Diameter Evolution at Different Tensions and Tension Fluctuation Coefficients

**Figure 5:**
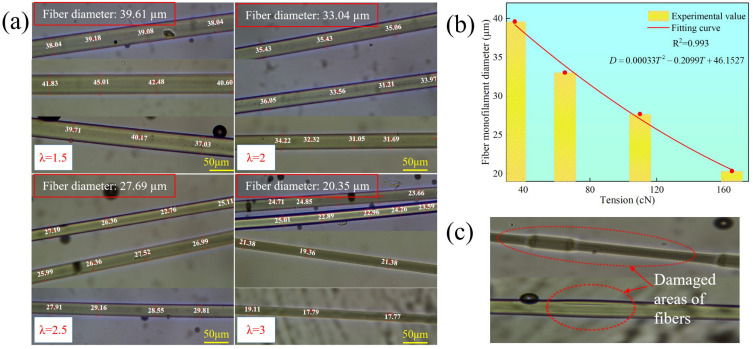
(**a**) Fiber monofilament diameter under different tensions. (**b**) Change rule of fiber monofilament diameter under different tensions. (**c**) Appearance changes in fiber monofilament under high tension fluctuations.

The fiber monofilament diameter plays a crucial role in the performance of the subsequent products. Figure 5a illustrates the variation in fiber monofilament diameter under different tension levels during the spinning process. As shown in Figure 5a, at a tension of 34 cN, the corresponding monofilament diameter is 39.61 µm; at a tension of 65 cN, the diameter is 33.04 µm; at 111 cN, it is 27.69 µm; and at 164 cN, the diameter is 20.35 µm. The results indicate that as the tension increases, the monofilament diameter decreases. A function relationship between tension and monofilament diameter was derived from the experimental results, with the fitted curve shown in Figure 5b. Additionally, observations using a metallurgical microscope revealed the impact of tension fluctuations on the appearance of the fiber. It was found that when the average tension fluctuation coefficient was 18.18%, the measured fiber monofilament diameters were noticeably inconsistent, and abrupt diameter changes occurred. This phenomenon is attributed to significant tension fluctuations, which cause breakage, uneven yarn thickness, and the formation of microfibers, as shown in Figure 5c.

### 3.2. Fiber Orientation Evolution at Different Tensions and Tension Fluctuation Coefficients

**Figure 6:**
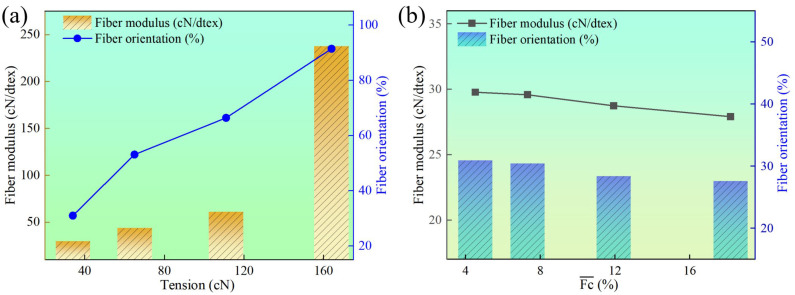
(**a**) Effect of the tension on fiber orientation. (**b**) Effect of the tension fluctuation on fiber orientation.

As shown in Figure 6a, during the spinning process, an increase in fiber tension results in a higher modulus, indicating that increasing the draw ratio leads to further alignment of the molecular chains in the fiber and enhanced intermolecular interactions, making the fiber less prone to deformation. At lower draw tensions, the polyester fiber experiences minimal tensile stress during spinning, resulting in less alignment of the macromolecular chains in the axial direction and consequently a lower degree of orientation. Conversely, as the draw tension increases, the tensile stress on the polyester fiber also increases, causing the macromolecules or aggregated structural units to align in the axial direction. When the draw tension is increased from 34 cN to 164 cN (from a draw ratio of 1.5 to 3.0), the modulus increases by nearly eight times, and the degree of orientation improves by nearly three times. These results indicate that increasing fiber tension rapidly enhances the degree of fiber orientation.

The effect of tension fluctuations on the modulus and degree of orientation of the fibers is illustrated in Figure 6b. As the tension fluctuations increase, both the modulus and the degree of orientation of the fibers exhibit a slight decrease. This decline is attributed to the uneven tension experienced by the fibers, which reduces the modulus and results in a downward trend in both the modulus and degree of orientation, indicating a corresponding decrease in the mechanical properties of the fibers. Specifically, compared to fibers with an average tension fluctuation coefficient of 4.51%, those with an average tension fluctuation coefficient of 18.18% showed a 7.42% reduction in modulus and a 10.78% decrease in degree of orientation. Therefore, during the spinning process, larger fluctuations in fiber tension negatively impact the degree of fiber orientation, leading to significant variations in fiber quality.

### 3.3. Fiber Crystallinity Evolution at Different Tensions and Tension Fluctuation Coefficients

**Figure 7:**
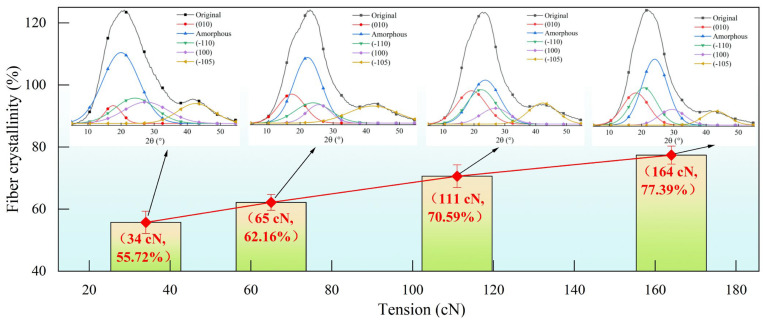
Effect of the tension on fiber crystallinity.

Figure 7 illustrates the effect of draw tension on the crystallinity of the fibers. As the draw tension during the spinning process increases, some loose molecules in the amorphous regions, which are loosely bonded to other molecules, gradually form new crystalline regions under the influence of draw stress. With the increase in draw tension, the macromolecules connected to the crystalline regions are also pulled out, contributing to the formation of extended chain crystals and resulting in a rapid increase in fiber crystallinity. When the draw tension is increased from 34 cN to 164 cN, the crystallinity of the fibers rises from 55.72% to 77.39%. Under different draw tensions, the polyester fibers exhibit strong primary X-ray diffraction peaks at 20.7°, indicating that the crystalline structure of the fibers does not undergo significant changes with increasing draw tension; however, the amorphous peaks narrow considerably, reflecting an increase in crystallinity. The enhancement in fiber crystallinity may be attributed to the increasing internal stress within the fibers as draw tension rises, which tightens the macromolecular chains and promotes the growth of characteristic diffraction planes. Additionally, there may be instances where the crystallites tilt along the draw direction under sustained tension, leading to fractures at locations within the fiber where crystallization defects exist, as shown in Figure 7. Therefore, the evolution of crystallinity in polyester fibers during the spinning process is closely related to the draw tension, and in practical production, adjusting the fiber tension can effectively regulate the crystallinity of the fibers.

**Figure 8:**
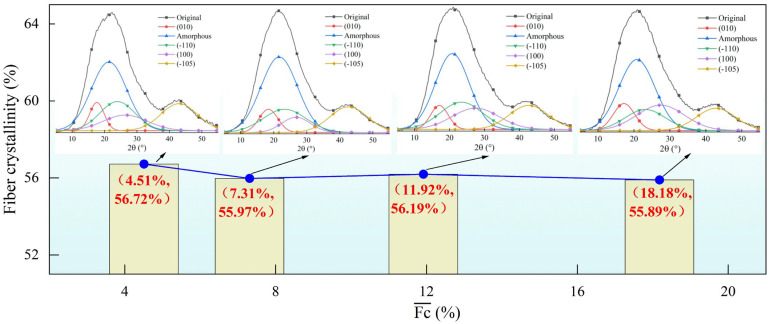
Effect of tension fluctuation on fiber crystallinity.

Figure 8 shows the effect of the tension fluctuation coefficient on fiber crystallinity. It can be observed that as the tension fluctuation increases, the overall crystallinity of the fibers decreases. From Figure 8, it is evident that as the tension fluctuation coefficient increases, the area of the amorphous peak slightly decreases, indicating that with a draw tension of 65 cN, the increase in tension fluctuation leads to a decreasing trend in fiber crystallinity.

### 3.4. Fiber Mechanical Properties Evolution at Different Tensions and Tension Fluctuation Coefficients

**Figure 9:**
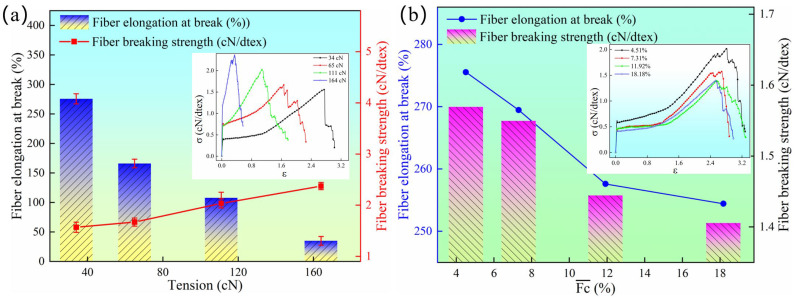
(**a**) Effect of tension fluctuation on the fiber mechanical properties. (**b**) Effect of the tension fluctuation on the fiber mechanical properties. The testing conditions were as follows: a gripping length of 100 mm, a stretching rate of 200 mm/min, with each test group consisting of 20 repetitions, and the average value was calculated from these measurements.

The draw tension of the fibers is a critical factor that cannot be overlooked during the thermal treatment process. Figure 9a illustrates the evolution of the fiber breaking strength and elongation at break under different draw tensions. As the draw tension increases, the breaking strength of the fibers gradually improves. At a draw tension of 34 cN, the breaking strength is 1.57 cN/dtex, while at a draw tension of 164 cN, the breaking strength reaches a maximum of 2.37 cN/dtex, representing an increase of 50.96%. From Figure 9a, it is also evident that as the draw tension continues to rise, there is a noticeable decreasing trend in the fiber elongation at break. Overall, at a draw tension of 34 cN, the maximum elongation at break can reach 275.55%, whereas at a draw tension of 164 cN, the minimum elongation at break is 34.95%.

Furthermore, Figure 9a indicates that the trends in breaking strength and elongation at break are inversely related as the draw tension increases from 34 cN to 164 cN. This phenomenon may be attributed to the tightening of the inter-fiber bonding forces with increasing tension, leading to a higher axial alignment of macromolecules within the fibers and an extension of the molecular chains. Consequently, during stretching, more macromolecules are subjected to stress, which macroscopically manifests as an increase in breaking strength and a decrease in elongation at break. This observation is consistent with the results presented in Figure 6.

Figure 9b illustrates the impact of tension fluctuations on the fiber breaking strength and elongation at break. As shown in Figure 9b, tension fluctuations significantly affect both the breaking strength and elongation at break of the fibers. With an increase in the tension fluctuation coefficient, both the breaking strength and elongation at break rapidly decrease. This is attributed to the large differences in fiber tension, leading to phenomena such as inter-fiber entanglement, overlapping, and uneven diameters between individual filaments within a yarn bundle. Compared to fibers with a tension fluctuation coefficient of 4.51%, fibers with a tension fluctuation coefficient of 18.18% exhibited a 10.36% decrease in breaking strength and an 8.14% reduction in elongation at break. These findings confirm that larger tension fluctuations during the spinning process result in a poorer mechanical performance of the fibers. In practical production, it is crucial to identify the underlying causes and reduce the tension fluctuation coefficient promptly.

## 4. Conclusions

This study investigates the effects of tension and tension fluctuations on fiber monofilament diameter, mechanical properties, orientation, and crystallinity by establishing a tension fluctuation coefficient during the spinning process. The experimental results demonstrate that there is a functional relationship between fiber tension and monofilament diameter, and that tension fluctuations can cause abrupt changes in monofilament diameter. Higher fiber tension leads to greater fiber orientation and crystallinity; however, when the average tension fluctuation coefficient increased by 13.67% during the spinning process, the fiber orientation decreased by 10.78%. As fiber tension increases, the breaking strength improves while the elongation at break decreases. Specifically, when the average tension fluctuation coefficient increased by 13.67%, the breaking strength decreased by 10.36% and the elongation at break decreased by 8.14%. These findings underscore the critical importance of maintaining stable tension during the spinning process to optimize the mechanical properties of polyester fibers. The ability to monitor tension fluctuations in real time can significantly enhance production efficiency and ensure consistent fiber quality, which is essential for various applications in the textile and materials industries. Moreover, this work can inform industrial practices and lead to the development of advanced polyester fibers with tailored properties for specific applications, such as high-performance textiles, industrial materials, and composite reinforcements.

## Data Availability

The original contributions presented in the study are included in the article, further inquiries can be directed to the corresponding authors.
